# Genetic dissection of an allotetraploid interspecific CSSLs guides interspecific genetics and breeding in cotton

**DOI:** 10.1186/s12864-020-06800-x

**Published:** 2020-06-26

**Authors:** De Zhu, Ximei Li, Zhiwei Wang, Chunyuan You, Xinhui Nie, Jie Sun, Xianlong Zhang, Dawei Zhang, Zhongxu Lin

**Affiliations:** 1 National Key Laboratory of Crop Genetic Improvement, College of Plant Sciences & Technology, Huazhong Agricultural University, Wuhan, 430070 Hubei China; 2 Shandong Key Laboratory of Dryland Farming Technology/Shandong Engineering Research Center of Germplasm Innovation and Utilization of Salt-tolerant Crops, College of Agronomy, Qingdao Agricultural University, Qingdao, 266109 Shandong China; 3 Shandong Peanut Research Institute, Qingdao, 266109 Shangdong China; 4 Cotton Research Institute, Shihezi Academy of Agriculture Science, Shihezi, Xinjiang, 832003 China; 5 Key Laboratory of Oasis Ecology Agricultural of Xinjiang Bingtuan, Agricultural College, Shihezi University, Shihezi, Xinjiang, 832003 China; 6 Institute of Industrial Crops, Xinjiang Academy of Agricultural Sciences, Urumqi, Xinjiang, 830091 China

**Keywords:** Cotton, Chromosome substituted segments lines (CSSLs), Quantitative trait loci (QTL), Whole genome re-sequencing, Cottonseed oil content (SOC)

## Abstract

**Background:**

The low genetic diversity of Upland cotton limits the potential for genetic improvement. Making full use of the genetic resources of Sea-island cotton will facilitate genetic improvement of widely cultivated Upland cotton varieties. The chromosome segments substitution lines (CSSLs) provide an ideal strategy for mapping quantitative trait loci (QTL) in interspecific hybridization.

**Results:**

In this study, a CSSL population was developed by PCR-based markers assisted selection (MAS), derived from the crossing and backcrossing of *Gossypium hirsutum* (Gh) and *G. barbadense* (Gb), firstly. Then, by whole genome re-sequencing, 11,653,661 high-quality single nucleotide polymorphisms (SNPs) were identified which ultimately constructed 1211 recombination chromosome introgression segments from Gb. The sequencing-based physical map provided more accurate introgressions than the PCR-based markers. By exploiting CSSLs with mutant morphological traits, the genes responding for leaf shape and fuzz-less mutation in the Gb were identified. Based on a high-resolution recombination bin map to uncover genetic loci determining the phenotypic variance between Gh and Gb, 64 QTLs were identified for 14 agronomic traits with an interval length of 158 kb to 27 Mb. Surprisingly, multiple alleles of Gb showed extremely high value in enhancing cottonseed oil content (SOC).

**Conclusions:**

This study provides guidance for studying interspecific inheritance, especially breeding researchers, for future studies using the traditional PCR-based molecular markers and high-throughput re-sequencing technology in the study of CSSLs. Available resources include candidate position for controlling cotton quality and quantitative traits, and excellent breeding materials. Collectively, our results provide insights into the genetic effects of Gb alleles on the Gh, and provide guidance for the utilization of Gb alleles in interspecific breeding.

## Background

Cotton is one of the most important cash crops, both as the leading natural fiber resource for the textile industry and an important oilseed crop. Approximately 50 species are present in the *Gossypium* spp., and only 4 species are cultivated worldwide: 2 are diploids (*G. herbaceum* and *G. arboreum*), 2 are tetraploids (*G. hirsutum* and *G. barbadense*). These two tetraploid (2n = 4x = 52) cotton species both share the common progenitors, which formed by a natural hybridization between A genome and D genome 1–2 million years ago [1–3]. The *G. hirsutum* (Gh), known as Upland cotton, contributed over 95% of cotton fiber yield by its wide adaptation and high yield [4, 5]. Because of the long process of domestication and selection bottlenecks, the elite Upland cotton has a narrow genetic base and limited genetic diversity [3]. This limitation could be a serious obstacle to improve the fiber quality and maintain continuity in genetic effectiveness [4]. While *G. barbadense* (Gb), also known as Sea-island cotton or long extra staple cotton, has excellent fiber quality, disease resistance but lower yield [6]. Introgression of interspecific favorable alleles to the Upland cotton can make full use of its high productivity, and it will be an ideal solution for cotton breeding [7, 8]. Although both of their genome sequence shared parts of the homology [9, 10], limited successes have been made in cotton interspecific breeding [6, 11]. Therefore, identifying, cloning, and utilizing beneficial allelic genes from the Gb will be important.

The primary segregating populations such as F_2_, BC_1_, have been widely used in genetic analysis for genetic map construction and quantitative trait loci (QTL) mapping. However, several disadvantages such as temporary nature and large deviation for evaluating the small-effect QTL limited their applications in the complex QTL analysis and cloning [12, 13]. In recent years, chromosome segment substitution lines (CSSLs), or referred as introgression lines (ILs), produced by crossing and backcrossing the donor and recipient parents by marker-assisted selection (MAS), provide a useful approach to resolve complex genome and QTL mapping [8]. Each of the CSSLs has one or few homozygous chromosome segments of donor genotype in the genetic background of the recurrent parent [14], which combines the advantages of the near-isogenic lines and backcross inbred lines. Through repeatedly planted in various locations or in different years, CSSLs helped to improve the accurate resolution of the genetic effects in the interspecific genomes [15–18]. Since the pioneering work in tomato [19], several interspecific introgression line libraries have been produced in many crops [20, 21]. Based on traditional molecular markers, such as restriction fragment length polymorphism (RFLP), amplified fragment length polymorphism (AFLP) and simple sequence repeat (SSR), a lot of QTL have been identified. However, limited by low genetic diversity and genetic map density, these molecular markers can identify only a few QTL and cover a wide region in the genome, which reduce the direct application of the QTL in breeding [22, 23].

In recent years, whole-genome re-sequencing technology has been widely used in population genetic analysis [24–26]. The high-throughput genotyping platform of SNP markers has significantly driven the process of genetic mapping and QTL identification [27–29]. Compared with the low density of traditional molecular markers, SNP markers significantly improve the genome coverage and QTL mapping accuracy. Multiple novel QTL for the important agronomic traits have been identified in multiple crops [30–32]. Moreover, high-resolution SNPs are a versatile tool to characterize the relationships between genes and importantly agronomic traits [33].

The prospect of widening the genetic diversity and improving the fiber quality of Upland cotton by accessing the exogenous genes has encouraged interspecific hybridization and introgression efforts for many years [6]. Stunning fiber quality of the Gb promotes it’s widely use in interspecific hybridization. Benefiting from widely range of variations shown in the progeny from Gh × Gb population, a large number of QTL related to multiple traits have been identified (https://www.cottonQTLdb.org). Moreover, some genes controlling specific characteristics of the Gb have been fine-mapped or cloned, such as open-bud floral buds [34], okra leaf [35–37], and naked seed mutant [38, 39]. Other wild *Gossypium* gene pools also provide a broad genetic diversity for Upland cotton [40–42]. However, none of them used high-throughput sequencing technology for analysis, which partly because there was no ultra-high density genetic map covering the entire genome or high-quality tetraploid cotton reference genome in the public domain. In the last a few years, spells above have been lifted in our lab [10].

Here, a set of interspecific CSSLs derived from a cross between *G. hirsutum* cv. ‘Emian22’ and *G. barbadense* acc. 3–79, were developed by using molecular marker selection. Next-generation sequencing technology was used to re-genotype all the lines and their parents by re-sequencing. The CSSLs were evaluated by using PCR-based markers and high-quality SNPs, resulting in a total of 480 introgression segments and 1211 recombination bins, respectively. Fourteen important agronomic traits including yield, fiber quality and oil content traits were measured in five environments to detect QTL. The influence of the Gb chromosome segments in the Gh background was investigated in this study.

## Result

### Evaluation of introgression chromosome recombination fragments in CSSLs

**Fig. 1:**
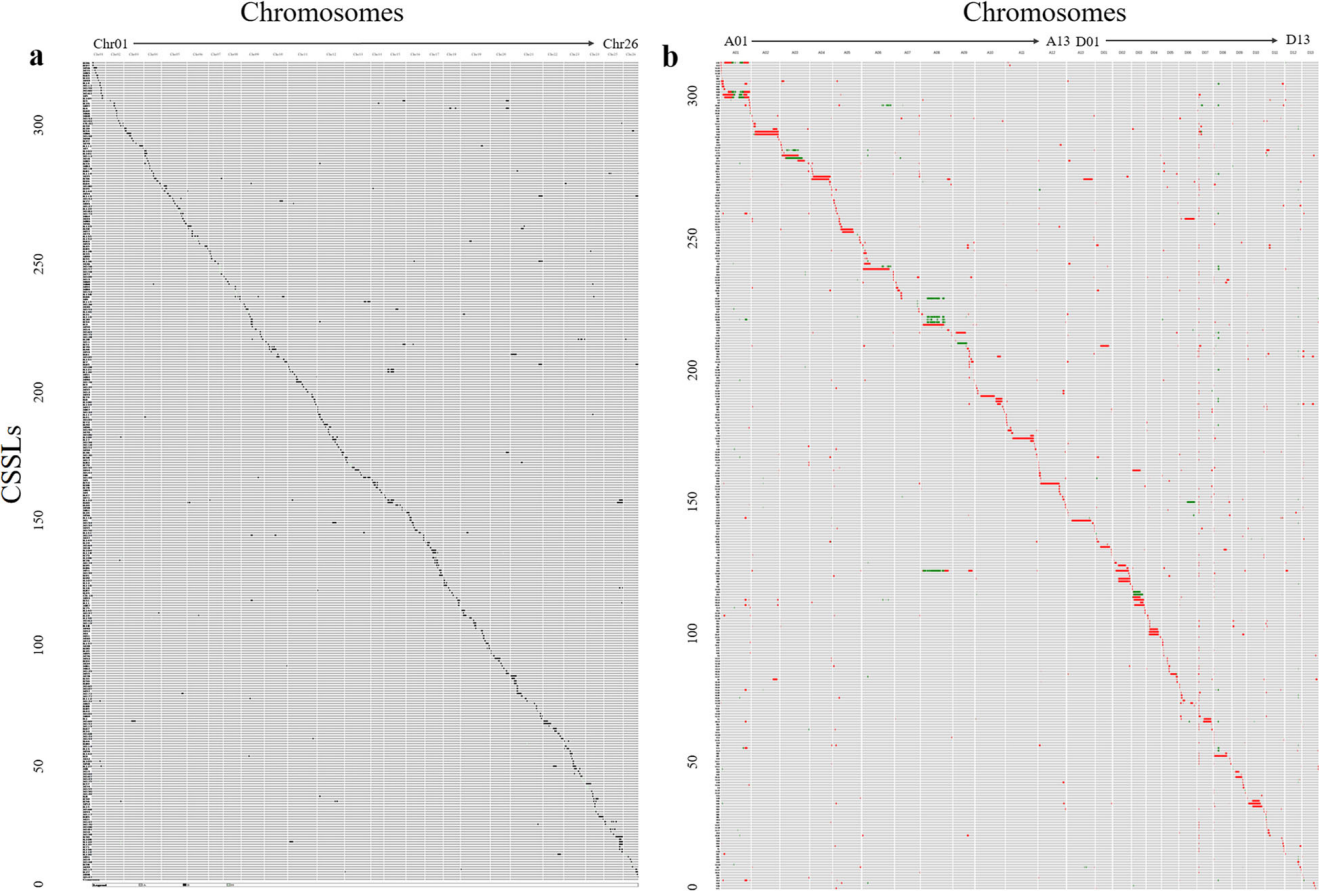
Distribution of introgression segments in the CSSLs on the 26 chromosomes. **a** Physical map was constructed by SSR markers; **b** Physical map was constructed by whole-genome re-sequencing SNPs. Each row indicates a CSSL, and each column represents a chromosome. The black and red squares denote the homozygous donor segments from Gb; the light-gray and green represent the heterozygous from Gb; the grey background represents the genetic background of the Gh

After several generations of self-pollinated, 515 markers were selected to evaluate the locations of introgression segments from donor parent in the lines with multi-segments again. Based on the genotypes of the molecular markers and the basis of the physical locations, the lengths and the locations of the introgression segments in each line were determined (Table 1), and a physical map was constructed (MM-map) (Fig. 1a). A total of 480 introgression segments were identified in the 325 CSSLs using SSR markers, with introgressions ranging from the least 10 ones on chromosome A03, D02 and D04 to the most 30 ones on chromosome D11. Among these, 222 lines carried one introgression segment despite the differences in lengths, and 103 lines were classified into the multi-segments group (Additional file 1: Table S1).

**Table 1:** Comparison of genetic map and physical map in the CSSLs

Chr.	Chromosome length		Number of markers		Average size		Number of segments		Coverage length		Coverage rate	
	MM-map (cM)	GR-map (Mb)	MM-map	GR-map	MM-map (marker/cM)	GR-map (SNPs/kb)	MM-map	GR-map	MM-map (cM)	GR-map (Mb)	MM-map	GR-map
A01	115.34	117.71	14	361,606	8.24	3.1	19	91	82.08	108.57	71.16%	92.24%
A02	147.16	108.05	18	604,209	8.18	5.6	19	40	76.08	105.69	51.70%	97.81%
A03	161.99	113.01	19	596,783	8.53	5.3	10	53	137.58	104.41	84.93%	92.39%
A04	140.78	85.11	18	466,455	7.82	5.5	20	41	123.75	82.41	87.90%	96.82%
A05	207.21	109.37	21	524,501	9.87	4.8	20	53	169.42	85.68	81.76%	78.34%
A06	172.09	124.01	19	662,304	9.06	5.3	16	46	120.19	114.98	69.84%	92.72%
A07	115.52	97.74	16	528,301	7.22	5.4	14	34	29.42	39.40	25.46%	40.31%
A08	141.97	122.33	20	673,976	7.10	5.5	13	60	63.98	120.00	45.06%	98.09%
A09	187.02	82.06	21	432,266	8.91	5.3	23	39	124.75	78.94	66.70%	96.19%
A10	185.70	114.80	21	616,965	8.84	5.4	16	22	163.96	100.14	88.29%	87.23%
A11	239.20	123.16	29	665,538	8.25	5.4	23	33	193.20	121.58	80.77%	98.72%
A12	222.31	107.62	26	600,282	8.55	5.6	29	42	202.90	100.35	91.27%	93.24%
A13	213.83	108.33	23	611,835	9.30	5.6	14	27	171.66	102.50	80.28%	94.62%
**At subgenome**	2250.12	1413.31	265	7,345,021	8.49	5.2	236	581	1658.96	1264.64	73.73%	89.48%
D01	178.95	63.18	22	331,752	8.13	5.3	17	27	124.09	53.84	69.34%	85.21%
D02	102.25	69.81	12	405,754	8.52	5.8	10	14	85.99	64.76	84.10%	92.76%
D03	145.67	52.68	17	302,944	8.57	5.8	20	27	132.24	52.67	90.78%	99.98%
D04	167.36	56.41	20	299,802	8.37	5.3	10	17	145.07	48.92	86.68%	86.73%
D05	243.38	62.90	28	295,927	8.69	4.7	22	35	196.42	51.82	80.70%	82.38%
D06	153.96	66.84	18	359,172	8.55	5.4	29	36	131.23	61.67	85.24%	92.26%
D07	152.94	59.23	18	308,614	8.50	5.2	14	126	129.79	56.28	84.86%	95.02%
D08	145.99	69.01	16	360,933	9.12	5.2	21	116	145.99	67.78	100.00%	98.22%
D09	174.52	52.80	20	290,463	8.73	5.5	25	29	158.55	44.98	90.85%	85.19%
D10	160.88	67.98	17	378,681	9.46	5.6	14	36	126.42	62.95	78.58%	92.61%
D11	265.89	72.91	32	321,124	8.31	4.4	30	62	209.45	32.85	78.77%	45.06%
D12	123.72	62.67	14	305,337	8.84	4.9	15	73	88.12	30.38	71.22%	48.48%
D13	136.87	63.32	16	348,137	8.55	5.5	17	32	120.31	29.40	87.90%	46.43%
**Dt subgenome**	2152.38	819.74	250	4,308,640	8.61	5.3	244	633	1793.67	658.29	83.33%	80.31%
**Total**	4402.50	2233.05	515	11,653,661	8.5	5.2	480	1211	3452.62	1922.94	78.42%	86.11%

MM-map: based on the genetic map constructed with molecular markers

GR-map: based on the physical map constructed by whole-genome resequencing

Based on SNPs from the sequencing data, 17,992 recombinant bins distributed on the 26 chromosomes were identified, which ultimately constructed 1211 recombination chromosome introgression segments from Gb in the 313 CSSLs (Fig. 1b and Additional file 2: Table S2). None chromosome introgression segments were detected in 10 lines in the CSSLs populations based on SNPs. The physical length of the introgression segments ranged from 97 kb to 104.23 Mb, with an average length of 4.43 Mb. Based on the physical map (GR-map), re-sequencing data significantly reduced the number of SSSLs, only 54 lines carried only one donor segment, and the lines with less than four segments just closed to half of the population (Additional file 1**:** Table S1). Significant difference of introgressions appeared in Dt-subgenome with 14 one on D02 and 126 ones on D07 (Table 1).

### Comparison of the genome coverage between SSR markers and SNPs

Based on the marker position of the genetic map, 6175.33 cM of the total length of the donor segments was counted by SSR markers, with 3462.62 cM of effective coverage length. The whole cotton genome coverage based on the genetic map was 78.42%, and At-subgenome had a lower coverage ratio of 73.73% compared with the 83.33% in Dt-subgenome. The lowest coverage was on chromosome A07 with only 25.46%, and the highest appeared in the Dt-subgenome with no missing on chromosome D08 (Table 1).

The physical map constructed by SNPs covered 2.24 times of the total length of the cotton genome (Additional file 3: Table S3), with 1922.93 Mb of effective coverage length and 86.11% whole genome coverage. Compared to the MM-map, GR-map had a higher percentage of coverage in At-subgenome (89.48% in At-subgenome vs 80.31% in Dt-subgenome). Although the coverage of 16 chromosomes exceeded 90%, there were still 4 chromosomes with coverage of less than 50%. Notably, chromosome A07 had the lowest coverage consistent with the MM-map result, and more than 98 CSSLs detected the same segment on the chromosome D07 located at 5.0–6.5 Mb.

### Phenotypic variation in CSSLs

Significant differences were observed between the parents across multiple traits and multiple environments, such as seed cotton weight per boll (BWT), lint percentage (LP), seed oil content (SOC) and all fiber quality traits. Fourteen traits were evaluated in five environments except that SI was just investigated in two environments (Additional file 4: Table S4 and Additional file 5: Table S5), and all traits showed a continuous distribution in the CSSLs. The broad-sense heritability (*H*^*2*^) was lower than 50% for the yield-related traits, indicating that they were easily affected by the environment (Additional file 6: Table S6). Higher *H*^*2*^ value of the lint percentage (LP) (76%), fiber length (FL) (77%) and SOC (87%) indicated that they were more affected by the associated genes coming from the Gb-genome. Fiber quality of Gb was outstanding in all environments, while the mediocre level of the fiber traits was observed in the lots of the CSSLs. Interestingly, recombination of the interspecific genomes also produced various fuzz fiber mutations with different densities and colors (Additional file 7: Figure S1). The N29 line produced fuzz-less phenotype similar to the Gb reported previously [10].

Positive and negative correlations between evaluated traits were calculated (Table 2). Plant height (PH) and first fruit branch height (FFBH) showed weak correlations with each other and with the yield-related traits (BWT and LP). But significant correlations were observed between fiber quality traits. Fiber length (FL) was significant positively correlated with fiber strength (FS) and fiber uniformity (FU), while negatively with micronaire value (MIC), fiber elongation (FEL), short fiber content (SFC) and fiber mature content (FM). The higher value of the SI followed the principle of negative correlation between yield and fiber quality, which may in turn increase of SOC.

**Table 2.**
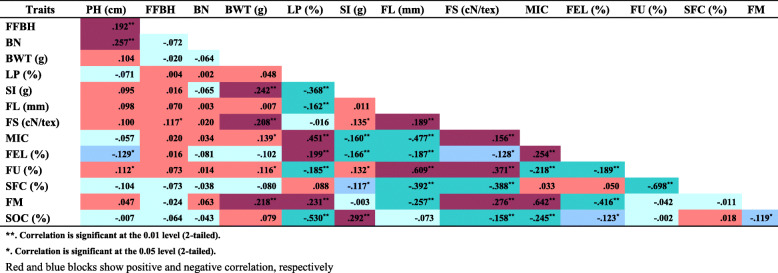
Correlation coefficients of 14 traits in the CSSLs over 5 environments.

**. Correlation is significant at the 0.01 level (2-tailed)

*. Correlation is significant at the 0.05 level (2-tailed)

Red and blue blocks show positive and negative correlation, respectively

### Genetic basis of the morphological mutation in the CSSLs

**Fig. 2:**
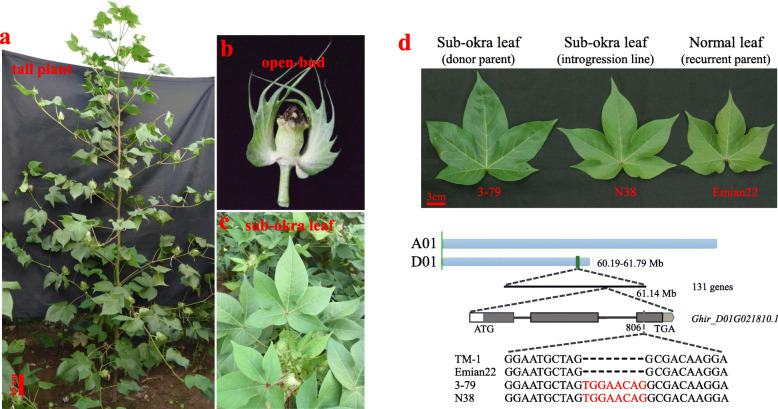
Some CSSLs showing morphological variations. **a** Significant tall mutant plant, **b** Open-bud mutant with stamen necrosis, **c** Sub-okra leaf, **d** Comparison of the *LMI1* gene structure in the CSSLs

Although the donor parent 3–79, the genetic standard of Sea-island cotton, had undergone artificial selection, cognitive of the plant height type for Sea-island cotton still appeared in the CSSLs (Fig. 2a). The “open-bud” floral buds phenotype was found during the flower development with the exposed stigma and dead anther (Fig. 2b). The associated marker BNL3479 located on chromosome D13 was similar to the former research (Additional file 8: Table S7) [34].

By using the high resolution of recombination segments, the iconic characteristic of the Gb, sub-okra leaf trait was identified in the CSSLs. Two nearby *KNOTTED1-LIKE HOMEOBOX I* transcription factors homologous to the *LATE MERISTEM IDENTITY1* (*LMI1*), *Ghir_D01G021810.1* and *Ghir_D01G021830.1*, were located near the 61.14 Mb on chromosome D01. An 8-bp deletion in the third exon of the gene *Ghir_D01G021810.1* showed the same mutation as reported previously (Fig. 2c and d) [37]. These examples showed that the high throughput detection methods could confirm an identified locus at a single gene-level resolution in this population.

### QTL mapping yield-related and fiber quality traits in the CSSLs

**Fig. 3:**
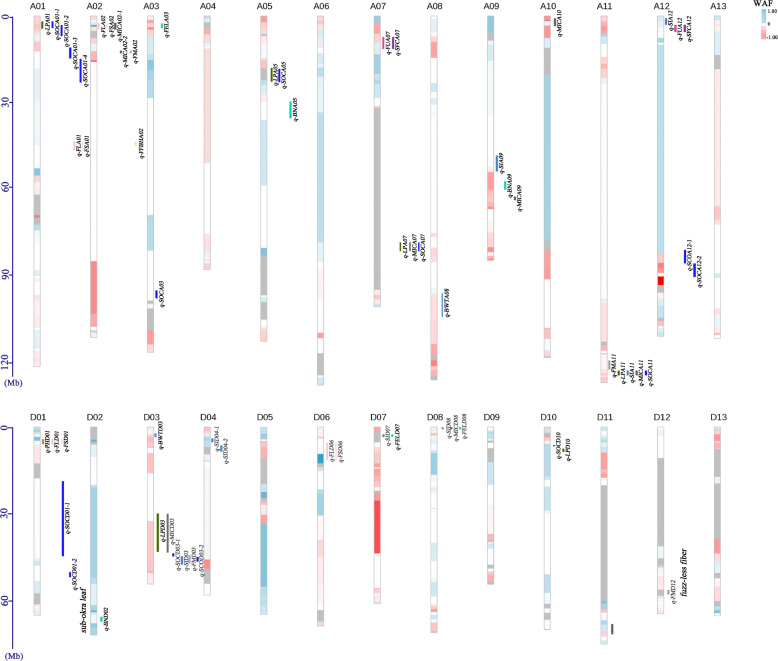
Chromosomal distribution of QTL and WAF value. Colored bars show the location of QTL. Red and blue indicate the additive effects from Gh and Gb, respectively; white and grey represent no effect and gap, respectively

To evaluate the valuable genetic loci of interspecific hybridization that are important in cotton breeding, QTL was mapped based on these CSSLs. The coverage fragments in the genome were divided into 620 blocks, with an average of 3.12 Mb ranging from 29 kb to 69.47 Mb (Additional file 9: Table S8). A total of 64 QTL for 14 traits were mapped on 20 chromosomes with 38 in At-subgenome and 26 in Dt-subgenome (Fig. 3 and Table 3). The phenotypic variation explained by each QTL ranged from 0.73 to 14.67%. There were 19 QTL for four yield-related traits (BN, BWT, LP and SI) and the favorite alleles were from the Gh background. All the QTL for BWT and LP had negative alleles from Gh background, suggesting that the Gh has been domesticated for high yield. While, two QTL had positive alleles for BN indicating that Gb also had the potential to increase yield production. A total of 28 QTL were detected for fiber quality traits, most of which (18/28) had positive alleles from Gb. Of these, completely co-localization was observed for FL and FS, indicating that there was a significant correlation between them. Eight QTL for MIC were detected on seven chromosomes which explained phenotypic variation ranging from 2.54 to 7.09%. Contrary to FEL and FU, the positive alleles of SFC and FM were contributed by Gh. Poor fiber quality phenotype in the CSSLs declined that the genetic recession has occurred in the interspecific hybrids between Gh and Gb.

**Table 3:** Summary of the QTL in the CSSLs

Traits	QTL	Blocks	Chr.	LOD	PVE (%)	Additive Effect	Block Interval		Publication
							Start	End	
**PH**	*q-PHD01*	block329	D01	3.33	4.78	1.02	3,186,046	4,556,068	
**FFBH**	*q-FFBHA02*	block67	A02	2.81	4.04	0.34	44,641,617	45,470,669	
**BN**	*q-BNA05*	block125	A05	3.67	4.86	0.97	31,292,912	34,892,387	
	*q-BNA09*	block224	A09	3.63	4.81	0.59	60,202,506	62,601,458	
	*q-LPD02*	block352	D02	2.59	3.41	−0.63	62,288,058	63,106,259	
**BWT**	*q-BWTA08*	block205	A08	3.04	3.97	−0.35	93,642,362	102,084,301	
	*q-BWTD03*	block358	D03	2.81	3.65	− 0.15	731,194	1,330,783	
**LP**	*q-LPA01*	block3	A01	6.40	6.95	−1.83	2,639,939	3,677,553	
	*q-LPA05*	block122	A05	4.57	4.89	−1.27	23,097,331	26,299,649	
	*q-LPA07*	block171	A07	3.29	2.99	−1.19	86,057,988	88,357,231	
	*q-LPA11*	block278	A11	5.10	5.48	−1.63	120,826,316	121,562,473	
	*q-LPD03*	block371	D03	8.39	9.24	−2.06	33,453,595	43,516,324	
	*q-LPD10*	block536	D11	2.62	2.37	−1.23	4,523,812	5,385,834	
**SI**	*q-SIA09*	block222	A09	4.79	2.48	0.31	54,020,891	58,352,764	
	*q-SIA11*	block278	A11	4.02	2.06	0.28	120,826,316	121,562,473	
	*q-SIA12*	block281	A12	4.31	2.21	0.33	12,595	809,856	
	*q-SID03*	block373	D03	3.38	1.73	0.36	44,469,168	47,084,844	
	*q-SID04–1*	block381	D04	17.64	10.03	1.23	2,305,353	3,131,469	
	*q-SID04–2*	block384	D04	24.46	14.67	−1.05	4,507,347	6,673,788	
	*q-SID07*	block446	D07	4.76	2.45	−0.08	5,062,647	5,765,634	
	*q-SID08*	block471	D08	3.85	1.98	−0.09	27,684	689,962	
**FL**	*q-FLA01*	block21	A01	2.53	2.89	0.48	51,165,194	53,482,140	
	*q-FLA02*	block59	A02	8.10	9.60	0.70	2,981,374	3,502,835	
	*q-FLD01*	block329	D01	3.93	4.53	0.51	3,186,046	4,556,068	
	*q-FLD06*	block428	D06	2.78	3.17	0.80	8,954,143	12,114,917	
**FS**	*q-FSA01*	block21	A01	2.53	2.89	0.48	51,165,194	53,482,140	
	*q-FSA02*	block59	A02	8.10	9.60	0.70	2,981,374	3,502,835	
	*q-FSD01*	block329	D01	3.93	4.53	0.51	3,186,046	4,556,068	
	*q-FSD06*	block428	D06	2.78	3.17	0.80	8,954,143	12,114,917	
**MIC**	*q-MICA02–1*	block59	A02	6.99	7.09	−0.20	2,981,374	3,502,835	
	*q-MICA02–2*	block64	A02	3.70	3.65	0.28	14,861,013	15,326,651	
	*q-MICA07*	block171	A07	2.70	2.44	−0.16	86,057,988	88,357,231	
	*q-MICA09*	block226	A09	6.40	6.46	0.24	63,998,867	64,921,374	
	*q-MICA10*	block237	A10	2.60	2.54	0.33	1,292,885	1,451,802	
	*q-MICA11*	block278	A11	3.46	3.42	−0.19	120,826,316	121,562,473	
	*q-MICD03*	block371	D03	3.04	2.99	−0.18	33,453,595	43,516,324	
	*q-MICD08*	block471	D08	4.29	4.27	0.07	27,684	689,962	
	*q-MICD11*	block580	D11	2.64	2.59	−0.14	71,643,126	72,910,318	
**FEL**	*q-FELA03*	block77	A03	4.84	6.91	0.62	1,173,846	2,339,035	
	*q-FELD07*	block446	D07	5.47	7.84	0.18	5,062,647	5,765,634	
	*q-FELD08*	block471	D08	3.16	4.45	0.17	27,684	689,962	Said et al.2015
**FU**	*q-FUA07*	block164	A07	3.01	5.57	0.36	8,903,355	12,793,020	
	*q-FUA12*	block282	A12	2.68	2.23	0.21	809,856	1,774,576	
**SFC**	*q-SFCA07*	block164	A07	3.15	4.27	−0.64	8,903,355	12,793,020	
	*q-SFCA12*	block282	A12	3.51	4.78	−0.43	809,856	1,774,576	
**FM**	*q-FMA02*	block64	A02	3.06	3.83	0.01	14,861,013	15,326,651	
	*q-FMA11*	block276	A11	2.94	3.83	−0.01	118,798,683	120,093,234	
	*q-FMD03*	block373	D03	2.53	3.21	−0.01	44,469,168	47,084,844	
	*q-FMD12*	block593	D12	2.94	3.83	−0.01	57,692,428	58,105,224	
**SOC**	*q-SOCA01–1*	block3	A01	21.66	5.84	2.32	2,639,939	3,677,553	
	*q-SOCA01–2*	block4	A01	3.14	0.73	−1.17	3,677,553	4,874,638	
	*q-SOCA01–3*	block12	A01	21.59	5.82	1.90	14,607,137	17,693,930	
	*q-SOCA01–4*	block13	A01	8.52	2.08	−1.60	17,693,930	28,547,783	
	*q-SOCA03*	block94	A03	4.62	1.09	−1.16	96,774,034	98,312,105	
	*q-SOCA05*	block122	A05	6.73	1.62	1.00	23,097,331	26,299,649	
	*q-SOCA07*	block171	A07	6.71	1.62	1.22	86,057,988	88,357,231	
	*q-SOCA11*	block277	A11	6.08	1.45	1.32	120,093,234	120,826,316	
	*q-SOCA12–1*	block293	A12	28.34	8.05	3.12	82,990,142	84,639,641	
	*q-SOCA12–2*	block294	A12	17.02	4.43	−2.85	84,639,641	86,335,298	Yu et al.2012
	*q-SOCD01–1*	block332	D01	34.76	10.40	−4.36	17,173,981	44,222,294	
	*q-SOCD01–2*	block333	D01	44.76	14.51	4.22	44,222,294	45,104,476	Yu et al.2012
	*q-SOCD03–1*	block372	D03	8.57	2.08	1.60	43,516,324	44,469,168	
	*q-SOCD03–2*	block374	D03	4.38	1.01	1.95	47,084,844	48,284,786	
	*q-SOCD10*	block535	D10	10.08	2.49	2.14	4,155,341	4,523,812	

### Genetic recession in the CSSLs

Genetic recession was a widespread phenomenon in the distant hybridization population. Fiber quality is one of the primary goals of cotton interspecific breeding. In this study, 7 lines with longer FL and 4 QTL for FL were identified in the CSSLs. Interestingly, two lines (N180 and R88) did not contain the QTL intervals, and two QTL intervals (on A01 and D06) also did not appear in the longer FL lines. The 13 fiber quality QTL identified in the single segment substitute lines (SSSLs) was inconsistent with the results of the same traits in this study except *q-FLA02* [10]. So, we designed a weight mean of additive effects of fiber quality (WAF) value to analyze the source of additive effect for minor-effect genetic loci. Based on the correlations among the fiber traits, the additive effect of the genome was calculated (Additional file 10: Table S9). As a result, At-subgenome from Gh showed a higher additive contribution to fiber quality, while D-subgenome from Gb showed opposite results (Additional file 11: Table S10). In the Gb genome, more than 80% regions of chromosome A012, D02 and D12 had an additive effect on fiber quality improvement (Fig. 3). In addition, there was no additive effect from Gb on chromosome D07. More than 90% regions of chromosome A11 showed the effect of Gh. Notably, the non-contribution effect for fiber quality in At-subgenome was signification higher than that in Dt-subgenome. Of these, both chromosome A08 and A12 from Gb or Gh had more than half of the regions contributing no effect for fiber improvement.

### QTL mapping for SOC and substitution mapping of QTL locus *q-SOCA01–1*

**Fig. 4:**
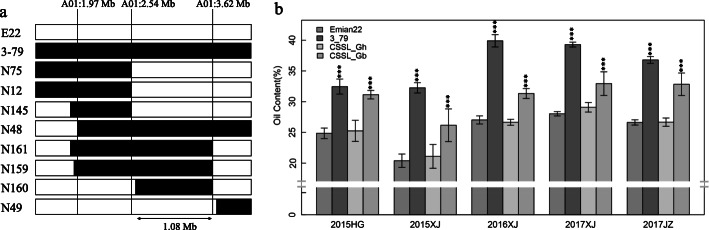
Substitution mapping of *q-SOC-1* using the 9 introgression lines (ILs) on chromosome. A01 **a** White and black represent the genotype of ‘Emian22’ and 3–79, respectively. **b** Seed oil content value are shown for five environments, the CSSL_Gh represent the background of Emian22(include the line of N75, N12, N49 and N145) and the CSSL_Gb represent the background of 3–79(include the line of N159, N160 and N161). One ANOVA analysis for two lines and Dunnett’s multiple comparison for multiple lines. ***. Indicated significantly different at the 0.001 level

Less concern of the SOC in Gb showed significant difference compared with the recurrent parent ‘Emian22’. A total of 12 lines showed extremely significant (*p* ≤ 0.001) and stable higher SOC than recurrent parent ‘Emian22’ (Additional file 12: Table S11), and 15 QTL were detected to be related to SOC using BLUPed data; of these QTL, 12 were firstly characterized and only two QTL for SOC have been reported previously in an interspecific population (Table 3) [43]. Fortunately, three SSSLs (N159, N160 and N161) contained the same block (block3) on chromosome A01, providing an excellent materials for further research. Compared with another 7 lines including the parents, these three lines showed extremely significant high SOC properties like the donor parent (Fig. 4). In the associated interval (block 3 ≈ 1.08 Mb), there were 69 and 70 annotated genes in the Gh reference genome TM-1 and Gb reference genome 3–79, respectively. A previously study showed that cottonseed oil accumulates rapidly at the middle-late stages (20 to 30 days post anthesis) [44]. Hence, we focused on the genes that are expressed in gradients in ovules with significantly higher expression levels than other tissues (root, stem, leaf and fiber) [10]. Among these genes, the Gene Ontology (GO) analysis indicated that only six were involved in fatty acid metabolism process in both genome (Additional file 13: Table S12). Unfortunately, it is not significant difference expression of these oil relate genes in ovule between Gh and Gb (Additional file 14: Figure S2). Intringuing, another gene, *Gbar_A01G002860.1*, encoding a predicted mitochondrial pyruvate dehydrogenase kinase (mtPDK), showed higher expression than its homologous gene *Ghir_A01G003150.1*. However, previous data from Marillia et al. reported that the seed-specific partial silencing of the mtPDK resulted in increased storage lipid accumulation in developing seeds [45]. Hence, this gene may play an important role in storage lipid accumulation in late developing stage of cotton seeds.

## Discussion

Cotton is the most important cash crop and contributes to more than 95% of natural textile fiber. Currently, improving the fiber quality by broadening the genetic basis of Upland cotton cultivars has become imperative. Construction of interspecific introgression lines can make full use of the superior fiber quality advantages of Gb on the basis of high yield of Gh, and also provide an ideal strategy for resolving the complex genome and QTL mapping. Several CSSLs with excellent agronomic traits than the Gh were found in this study, which can be directly applied to improve the fiber quality or SOC in cotton breeding.

### Development strategy of the cotton introgression lines

**Fig. 5:**
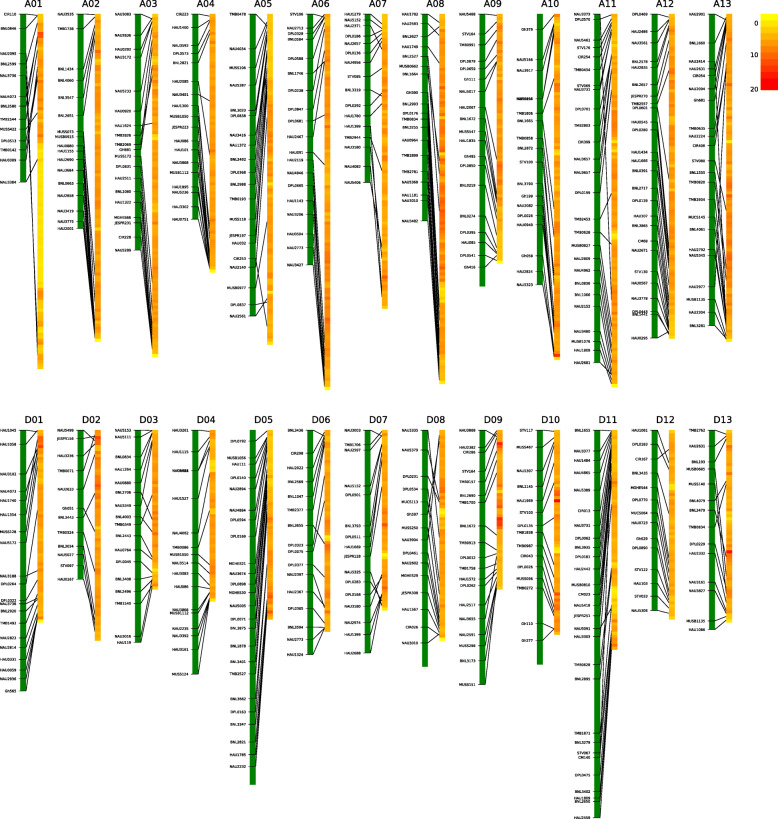
Comparison of genetic map and physical map in evaluating the CSSLs. Left and right show SSR markers and SNP markers position on the chromosome, respectively. Colors show the density of the SNPs. Marker’s position is linked by grey lines between two maps

The ideal introgression lines aim to product a series of SSSLs in which all the introgression segments cover the entire donor genome. High cost-effective ratio of PCR-based molecular markers makes it the first choice for tracking the introgression segments due to absence of high quality reference genomic sequence. In this study, a high-density interspecific genetic map between Gh and Gb cotton was constructed and updated. In the early stage, few markers were selected from the primary genetic map to survey introgressions in the early generations, and then new markers were engaged in the advance generations with only targeted region selection after updating the high-density linkage map, which could be significantly reduce the workload during the development of the ILs population. However, identification of false or missing segments cannot be avoided. As a result, a wide range of gaps were found in At-subgenome by aligning the reference genome, especially on chromosome A01, A02, A03 and A06 (Fig. 5). Non-collinear arrangement and clustering of the SSR markers on the physical map significantly reduced the coverage of the genome. Significant clustering of SSR markers appeared at the both ends of multiple chromosomes, such as A02, A03, A06 and A08, which was consisted with that a lot of lines carried a long fragment detected by several sequential markers.

Despite that, the high-density linkage map constructed by our lab still showed a certain advantage in this study. Several SSSLs were confirmed by genome re-sequencing which were identified by PCR-based molecular markers.

### High-throughput genotyping technology provides highly reliable introgression

The whole genome re-sequencing technology provides a strategy to understand the entire genomic variations after having a high quality reference genomic sequence, which could help to improve the detection of the donor segments in the whole genome. In this study, the CSSLs were genotyped using next-generation sequencing following the project of the reference genome [10], and an ultrahigh-quality physical map by SNPs was constructed, which was a pioneer study to use this strategy for genotyping CSSLs in cotton. As a result, lots of small segments were newly detected by sequencing, which significantly reduced the number of corresponding chromosomes and candidate confidence intervals for the associated traits. Some segments containing the candidate genes cannot be effectively assessed by SSR markers, although these markers were closely linked with the target trait. For example, the sub-okra leaf shape gene was detected by whole genome re-sequencing, while the MM-map only showed that there was a marker associated with this trait. In this study, none introgression segments were detected in 10 lines by SNPs. The reason is that the introgression fragments in these lines identified by SSR markers are less than 100 kb in length, which were marked as ‘not available’ and filtered. Besides homozygous introgressions, a number of heterozygous fragments were detected on chromosome A01 and A08 after a few rounds of self-fertilization. For example, line R28 carried the heterozygous fragment covering almost the entire chromosome A08, and line R126 carried a wide range of heterozygous fragments on different chromosomes which may result in colorful phenotype of the fuzz fiber (Additional file 7: Figure S1). Consistent with the previous reports [46, 47], we speculate that this may be related to the interspecific segregation distortion.

Based on the above results, we conclude that construction of an ideal introgression population can follow this strategy: (1) PCR markers from high-density genetic map are used to construct the primary introgression lines in the primary generations to decrease the cost; (2) all the lines are genotyped by high-throughput re-sequencing technology to accurately identify the introgression segments; (3) further backcrossing of the lines carrying more than one segment will be performed to achieve the purpose of constructing SSSLs.

### CSSLs constructed a platform for resolving the polygene hypothesis

Quantitative traits are usually regulated by multiple minor-efficient genetic loci, which modified by the genetic and external environments [48]. Different QTL for fiber traits were detected between the SSSLs [10] and the whole lines (this study), indicating that the genetic loci for superior fiber quality of the Gb was controlled by multiple genes and dispersed on different chromosomes. A notable evidence appeared in this study was that the CSSLs (N180 and R88) carried multiple donor fragments but did not contain the QTL loci, which means that the genetic effects of these introgression fragments were low enough to be detected as a major QTL. Consistent with the previous study of the introgression population, we aimed at dissecting the donor genome by MAS in this study. However, this strategy may undermine the genetic pattern of quantitative traits such as fiber quality traits, which commonly regulated by multiple genes at different development stages [49]. Hundreds of high expression levels of genes during fiber development also illustrated this view [10]. These co-effector genes derived from Gb donor were segmented and dispersed in different lines, which blocked the regulatory relationship between them. As a result, we summarized that fewer introgression fragments in the SSSLs may effectively block the interaction between different genetic backgrounds and between loci on different chromosomes, which facilitated the detection of the minor-efficient genetic loci [10]. While more introgression segments and higher genomic coverage, especially the long fragments, the noise and epistasis effects were effectively reduced, which improved the reliability of identifying major and stronger effective loci that can be directly applied into breeding in the future. Similar conclusion in previous reports just had a brief description [31, 50, 51]. However, correlations between phenotypes may indicate that complex quantitative traits are controlled by same gene or closely linked genes. Many fiber quality QTL were detected in the interval of block 59 in this study, which indicated that there still existed the single major genetic locus for fiber quality in the Gb genome. Therefore, we can conclude that the genetic locus controlling fiber quality in the Gb genome is the interaction of the major gene with the minor-effect polygenic loci scattered on different chromosomes, and the future breeding for improving fiber quality should try to pyramid more beneficial factors.

### Sea-island cotton as an excellent resource for improving cottonseed oil content

Cottonseed oil has a large amount of unsaturated fatty acids [52]. Several lines with higher SOC were identified which could be directly used in oil improvement breeding, connecting with the higher value (87%) of the broad-sense heritability. Multiple QTL for SOC were detected on different chromosomes in this population, which suggested that there should be a network between genes controlling the SOC in the Gb. These results indicate that Sea-island cotton has a high potential in improving the SOC of Gh. In this study, we predicted that a PDK gene may regulated the SOC in Gb, which indicated that the growth advantages of Sea-island cotton may have a more positive influence on regulating other traits than Upland cotton. Complex fatty acid metabolism pathway and the diversity of lipid compositions increase the difficulty to propose the candidate genes in the confidence intervals. However, based on the genomic annotation variation combining with transcriptome and metabolome analysis, the relevant information of the lipid biosynthesis is sufficient to identify candidate genes in the future, which have been proved to be feasible [12, 53].

## Conclusions

Plant breeding aims to integrate multiple desirable traits to obtain elite varieties. Introgression between different species is a key process to broaden the genetic basis of the breeding materials. In this study, we developed a CSSLs population carrying introgression segments from Gb in the Gh background. The whole-genome re-sequencing technology was applied to study the CSSLs to construct the high-quality physical map for each line, which provided more accurate introgression than in the map constructed by SSR markers. A total of 64 QTL were mapped for 14 agronomic traits and favorite Gb alleles for fiber quality were identified. Importantly, novel Gb alleles for increasing SOC were found. Our study not only offered guides for future molecular breeding to increase fiber quality and SOC, but also provided a reference basis for fine-mapping and map-based cloning genes to genetic improvement of Upland cotton.

## Methods

### CSSLs development

In this study, ‘Emian22’ (*G. hirsutum*) and ‘3–79’ (*G. barbadense*), were used to develop CSSLs. ‘Emian22’ is an upland cotton cultivar with high yield and moderate fiber quality in Hubei Province. And the ‘3–79’ is a genetic and cytogenetic standard line for *G. barbadense* with super fiber quality and high resistance to *Verticillium wilt*. ‘Emian22’ and ‘3–79’ are public available materials and have been kept in our laboratory nearly twenty years. The construction process of this CSSLs population has been brief described in the previous article [10]. In 2006, after four rounds of successive backcrossing, 254 whole-genomic SSR markers were selected to the whole-genome surveying 221 BC_4_ lines [54] (Additional file 15: Figure S3). The 82 BC_4_ plants covering the whole donor cotton genome were selected to be further backcrossed with ‘Emian22’, while some of these individuals were selected to be self-pollinated to produce BC_4_F_2_. In 2007, target regions were genotyped using the corresponding polymorphic markers in 1686 individual plants derived from 1028 BC_4_F_2_ and 658 BC_5_F_1_ individual plants. A total of 302 individuals out of them containing less than five, short chromosome segments and possibly covering the donor genome were selected, including 128 individuals with only one donor segment (Additional file 16**:** Figure S4). In 2008, 515 markers selected from the updated high-density linkage map [55], were used for re-evaluating the plants. About 312 individuals were selected, of which 162 individuals had less than three donor segments (Additional file 17: Figure S5). The plants having only one donor segment were self-pollinated to produce the homozygous CSSLs, and the others were continually backcrossed with ‘Emian22’ to produce the advanced backcrossing generation. In the same way in 2009, corresponding polymorphic markers were executed to identify the target segment in all the lines, including the self-pollinated lines. About 336 individuals containing the target region were selected, including 60 plants with only one donor segment (Additional file 18: Figure S6). In the subsequent process, same steps were executed to select the plants with the target segments. Until 2011, 337 individuals were obtained with 279 plants having less than three target segments, of which 151 plants having only one donor segment (Additional file 19: Figure S7). After two rounds of self-fertilization to ensure the homozygous genotype, a set of 325 CSSLs including 177 SSSLs were ultimately obtained.

### Phenotype evaluation

All the CSSLs with their parents were planted in two replicated plots at three different locations which are authorized by local governments: Huanggang (HG), Hubei province and Shihezi (SHZ), Xinjiang province in 2015; Shihezi in 2016; Jingzhou (JZ), Hubei Province and Shihezi in 2017. Field management essentially followed the local agricultural practices. PH, FFBH, and BN were evaluated at blooming stage, including the morphology of the plants (leaf and flower). Twenty bolls from each line were hand-harvested from the internal middle parts of the plants at the mature stage in every year. Yield-related traits, such as BWT, LP, SI, were tested in this CSSLs. And seven fiber quality traits were investigated including FL, FS, MIC, FU, FEL, SFC and FM. The seed phenotypes were scored based on visual inspection; meanwhile, at least 10 g delinted seeds were used to measure for SOC by low field pulsed nuclear magnetic resonance apparatus (NMR) analyzer on a NM-12 (Niumai Analytical Instrument Corporation, China). Best linear unbiased predictions (BLUPs) with broad sense heritability (*H*^*2*^) were used to estimate phenotypic traits across all five environments in R package. Pearson correlation coefficients were calculated to analyse the relationship between traits using BLUPed data by SPSS 17.0 software (SPSS Inc., Chicago, IL, USA).

### Estimating the introgression segments in CSSLs using SSR markers

Total genomic DNA of the CSSLs and their parents was extracted from the fresh young leaves at seeding stage using modified CTAB method [56]. A total of 515 SSR markers selected from the high-density interspecific genetic map were used to genotype the CSSLs. The length of Gb introgression segment was estimated by the graphical genotype of the markers. If one marker has the same genotype as the donor parent, this line is considered to carry the introduced fragment from donor parent at this genetic position; otherwise, the genetic background will be considered to be the same as the recipient parent. A segment flanked by two markers with genotype DD, DR, RR, were considered to be 100, 50, 0% of donor type, respectively (Additional file 20: Figure S8). The “D” and “R” represent the donor and recipient genotype, respectively. Thus, the length of the introgression segment was estimated to be the total length of the DD length and two half of DR length [31].

### Identification of SNPs and introgression segments in the CSSLs

The CSSLs population was cultivated in the field in Wuhan, China, in 2017. Leaf tissues were collected for plant genome DNA extraction with the Plant Genome Extraction Kit (TIANGEN Biotech). The 177 SSSLs with the parents have been sequenced by Wang et al. [10]. The other 145 CSSLs were sequenced on the same Illumina HiSeq platform with at least 6× coverage (pair-end 150 bp; Additional file 21: Table S13). Meanwhile, the Gh parent line ‘Emian22’ was deep sequenced with 60× coverage. To redo SNP calling, all the clean sequencing reads were mapped on the *G. hirsutum* reference TM-1 genome using BWA software version 0.7.10 and SNPs were called using GATK software with previously reported method [10].

The CSSLs may had large introduced fragment at the Chromosome recombination interval, so the bin map could be a better strategy to instead consecutive SNPs. A slightly modified sliding windows approach [57] was applied to identify the donor segments from Gb (Additional file 22: Figure S9). Firstly, a total of 11,653,661 SNPs and an average of 5.3 per kb were detected between Gh and Gb, and used to construct the bin. Then, all the alleles represented by SNPs in each CSSL were filtered using SNPs from both parents. And only those having the same allele as one of the parents were retained. The genotype of each window was called with a window size of 50 kb and step size of 5 kb. The ratio of SNPs in the window was calculated (> 80% of SNPs had one parental genotype, the window was called as homozygous of one parent; otherwise, the window was called as heterozygous). Determination of the recombination breakpoints and construction of the bins were performed as described by Han et al. [57]. The regions between two adjacent bins with same genotypes less than 100 kb were defined as the same bin, and bins of less than 100 kb in length were filtered. The recombinant donor chromosome segments for each CSSL were constructed based on the recombinant bins.

### QTL mapping and weight mean of additive effects of fiber quality evaluation

To identify the QTL, the Gb introgression segments were divided into several non-overlapping blocks (Additional file 23: Figure S10), ensuring each line carries as smaller overlapping chromosome region as possible. The BLUPed data of the five environments was used as the response variations of the 14 traits. QTL mapping and additive effect calculation were performed using RSETP-LRT-ADD mapping method with QTL IciMapping V4.0 software [58]. The block interval was used as the QTL location, and QTL was named based on the rules of the reporting in the *Rosaceae* (recommendations for standard QTL nomenclature and reporting in the *Rosaceae* 2014). To obtain potential candidate genes, the annotated genes were identified for a Gene Ontology (GO) analysis and the transcription profiles for different tissues of TM-1 and 3–79 were employed as a reference [10].

Based on the QTL mapping results, the additive effect of all the fiber traits were calculated. Contributions of the Gb to the fiber quality in the Gh background were estimated using a weight mean model. Based on the correlations between the fiber traits and the broad sense heritability, the WAF model was described by the following formula: *t* represents the fiber quality traits, *Add*_*t*_ is the value of additive effects for each block, *r*_*t*_ is the value of positive correlation coefficient and *H*^*2*^_*t*_ represents the broad sense heritability of the related trait. The distribution of the WAF on chromosome was calculated based on the blocks interval.

$$ WAF=\frac{\sum {Add}_t{r}_t{H}^2t}{\sum {r}_t{H}^2t} $$

## Supplementary information

**Additional file 1: Table S1.** Segments carried by CSSLs.xlsx

**Additional file 2: Table S2.** Summary of introgression segments.xlsx

**Additional file 3: Table S3.** Summary of the segments in the CSSLs.xlsx

**Additional file 4: Table S4.** Description of investigated traits in CSSLs.xlsx

**Additional file 5: Table S5.** The phenotypic data of CSSLs.xlsx

**Additional file 6: Table S6.** Broad-sense heritability (*H*^*2*^) of 14 traits in the CSSLs.xlsx

**Additional file 7: Figure S1.** The fuzz fiber phenotypes in the CSSLs with their parent lines. TIFF

**Additional file 8: Table S7.** Morphological characteristics of specific introgression lines.xlsx

**Additional file 9: Table S8.** Summary of the blocks in the genome.xlsx

**Additional file 10: Table S9.** Additive effects of the fiber length with the positive traits.xlsx

**Additional file 11: Table S10.** Summary of the Weight mean of fiber Additive effects on chromosome.xlsx

**Additional file 12: Table S11.** Yield-related and fiber quality traits of specific CSSLs.xlsx

**Additional file 13: Table S12.** GO enrichment analysis of genes in the candidate chromosome region.xlsx

**Additional file 14: Figure S2.** Transcript profiles of promising genes for root, stem, leaf, fiber and ovule between Emian22 and 3-79.

**Additional file 15: Figure S3.** Summary of the introgression segments of the BC4 generation with 221 individuals in 2006.

**Additional file 16: Figure S4.** Summary of the introgression segments of 302 individuals in 2007. TIFF

**Additional file 17: Figure S5.** Summary of the introgression segments of 312 individuals in 2008. TIFF

**Additional file 18: Figure S6.** Summary of the introgression segments of 336 individuals in 2009. TIFF

**Additional file 19: Figure S7.** Summary of the introgression segments of 337 individuals in 2010. TIFF

**Additional file 20: Figure S8.** Example of chromosome introduction fragments evaluated by SSR markers. A. Genotype calling based on the graphic of SSR markers on the PAGE. The “DD” and “RR” represent the donor and recipient parent, respectively. B. Introgression fragments evaluating based on the genotype of two near markers: “DD” represents 100% (Marker2 and Marker3); “DR” represents 50% (Marker4 and Marker5); “RR” represents 0% (Marker 5 and Marker6). TIFF

**Additional file 21: Table S13.** Summary of DNA sequencing data for CSSLs.xlsx

**Additional file 22: Figure S9.** An overview of the introgression segment identification protocol. A. Schematic diagram on identification of chromosome introduced fragment in CSSLs. First, all of the CSSLs and their parents were sequenced on an Illumina HiSeq platform to produce the genome sequence. All clean data were mapped to the *G.hirsutum* (TM-1) genome using BWA software and the unique mapping data were retained for further analysis. Then GATK software were applied to identify the SNPs based on the criteria:(1) the quality of SNPs should be over 100; (2) each SNP was supported by at least five reads; and (3) the adjacent SNPs should have a distance of at least 10 bp. To identify the introgression segments in the CSSLs, the SNPs between parents were selected. And a modified sliding-window approach was applied to identify the donor segments from Gb. This approach has been described very clearly by Han et al [57]. All the alleles represented by SNPs in each CSSL were filtered using SNPs from both parents. A bin map was constructed based on the genotype results of the window and consecutive bins with the same genotype were combined into same segments. B. Example of the Genotype calling based on the ratio of the SNPs in the window(>80% of SNPs had one parental genotype, the window was called as homozygous of one parent; otherwise, the window was called as heterozygous). TIFF

**Additional file 23: Figure S10.** Example diagram of block partition. (A) The diagram show the principle of block partition; (B) The CSSLs carried the introgression segments on the endpoint of the chromosome A01; (C) First five blocks on the chromosome A01. TIFF

## Data Availability

The clean raw sequencing data in this manuscript have been deposited in NCBI Sequence Read Archive under accession number PRJNA433615 and PRJNA543759.

## Abbreviations

AFLP: Amplified fragment length polymorphism
BLUPs: Best linear unbiased predictions
BN: Boll number per plant
BWT: Weight per boll
CSSLs: Chromosome segments substitution lines
FEL: Fiber elongation
FFBH: First fruit branch height
FL: Fiber length
FM: Fiber mature content
FS: Fiber strength
FU: Fiber uniformity
Gh: *Gossypium hirsutum*
Gb: *G. barbadense*
GO: Gene Ontology
LP: Lint percentage
MAS: Markers assisted selection
MIC: Micronaire value
NMR: Nuclear magnetic resonance apparatus
PH: Plant height
QTL: Quantitative trait loci
RFLP: Fragment length polymorphism
SFC: Short fiber content
SNPs: Single nucleotide polymorphisms
SI: Seed index
SOC: Seed oil content
SSR: Simple sequence repeat
SSSLs: Single segment substitute lines (SSSLs)
WAF: Weight mean of additive effects of fiber quality
