# Supplementary figures and images for: Genetic dissection of an allotetraploid interspecific CSSLs guides interspecific genetics and breeding in cotton
Source: BMC Genomics. 2020 Jun 26;21:431. doi: 10.1186/s12864-020-06800-x (PMC7318736; doi:10.1186/s12864-020-06800-x)

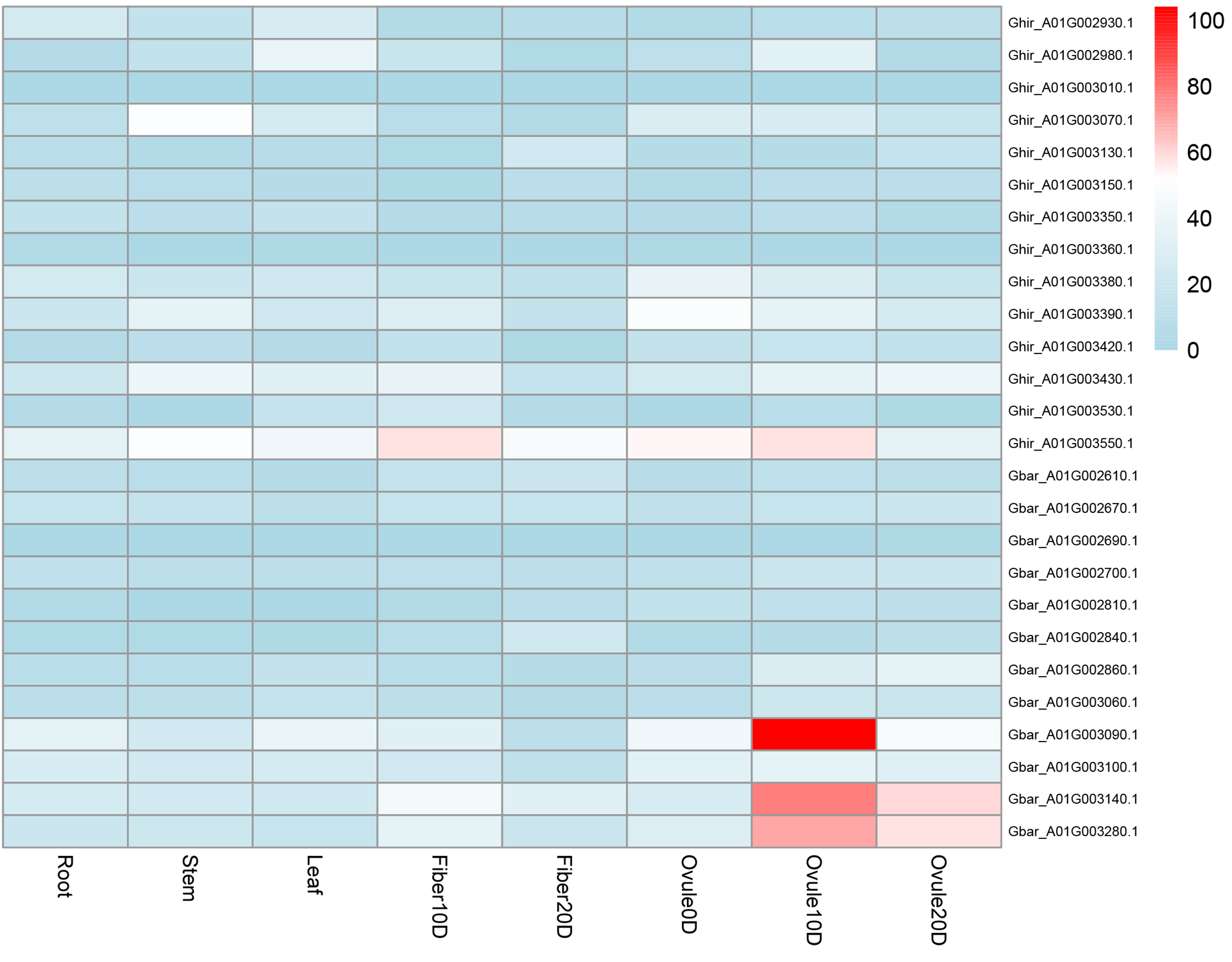

Supplement: Supplementary file 14 — Additional file 14: Figure S2. Transcript profiles of promising genes for root, stem, leaf, fiber and ovule between Emian22 and 3-79. [file 12864_2020_6800_MOESM14_ESM.tif]

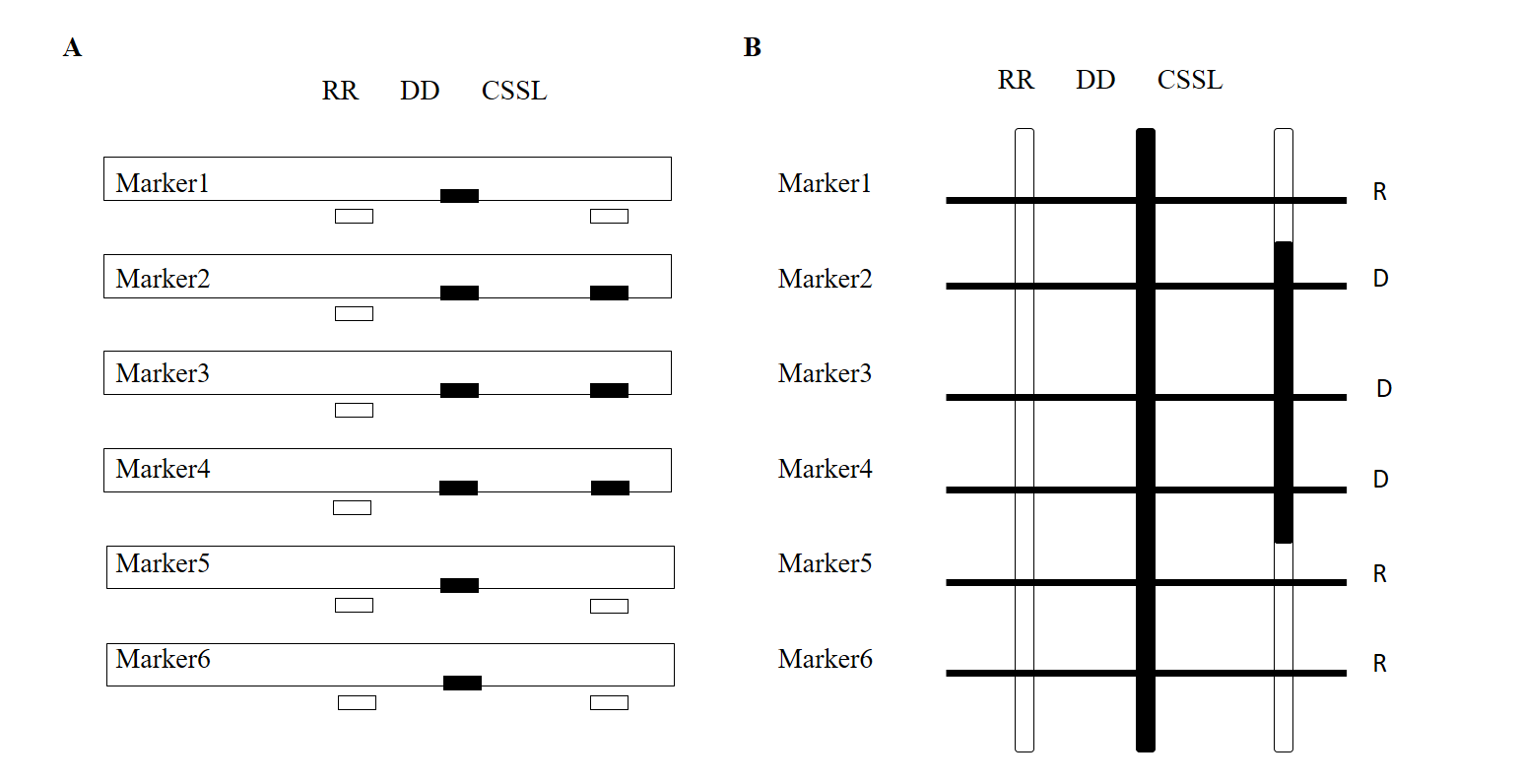

Supplement: Supplementary file 20 — Additional file 20: Figure S8. Example of chromosome introduction fragments evaluated by SSR markers. A. Genotype calling based on the graphic of SSR markers on the PAGE. The “DD” and “RR” represent the donor and recipient parent, respectively. B. Introgression fragments evaluating based on the genotype of two near markers: “DD” represents 100% (Marker2 and Marker3); “DR” represents 50% (Marker4 and Marker5); “RR” represents 0% (Marker 5 and Marker6). TIFF [file 12864_2020_6800_MOESM20_ESM.tif]

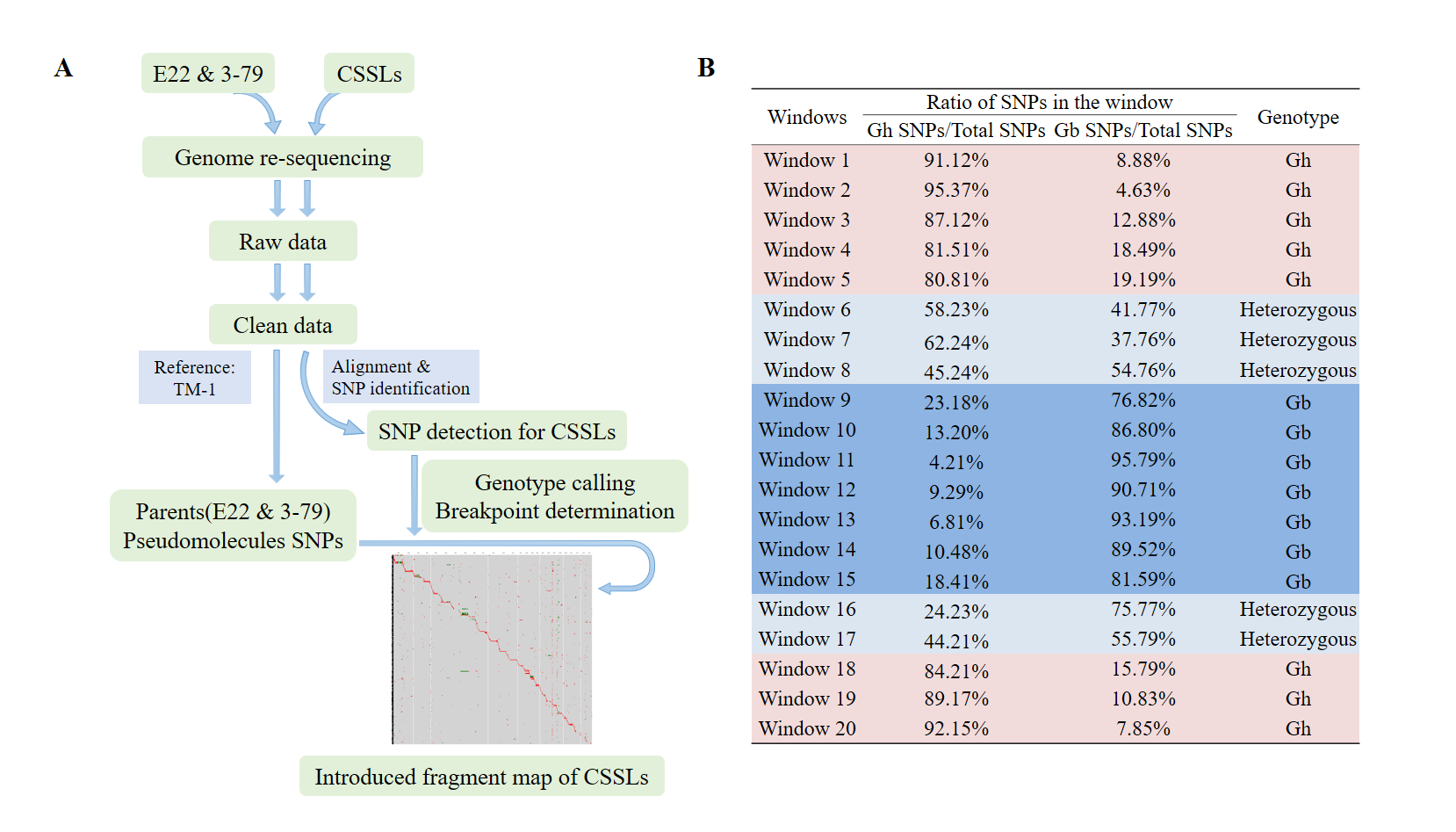

Supplement: Supplementary file 22 — Additional file 22: Figure S9. An overview of the introgression segment identification protocol. A. Schematic diagram on identification of chromosome introduced fragment in CSSLs. First, all of the CSSLs and their parents were sequenced on an Illumina HiSeq platform to produce the genome sequence. All clean data were mapped to the G.hirsutum (TM-1) genome using BWA software and the unique mapping data were retained for further analysis. Then GATK software were applied to identify the SNPs based on the criteria:(1) the quality of SNPs should be over 100; (2) each SNP was supported by at least five reads; and (3) the adjacent SNPs should have a distance of at least 10 bp. To identify the introgression segments in the CSSLs, the SNPs between parents were selected. And a modified sliding-window approach was applied to identify the donor segments from Gb. This approach has been described very clearly by Han et al [57]. All the alleles represented by SNPs in each CSSL were filtered using SNPs from both parents. A bin map was constructed based on the genotype results of the window and consecutive bins with the same genotype were combined into same segments. B. Example of the Genotype calling based on the ratio of the SNPs in the window(>80% of SNPs had one parental genotype, the window was called as homozygous of one parent; otherwise, the window was called as heterozygous). TIFF [file 12864_2020_6800_MOESM22_ESM.tif]

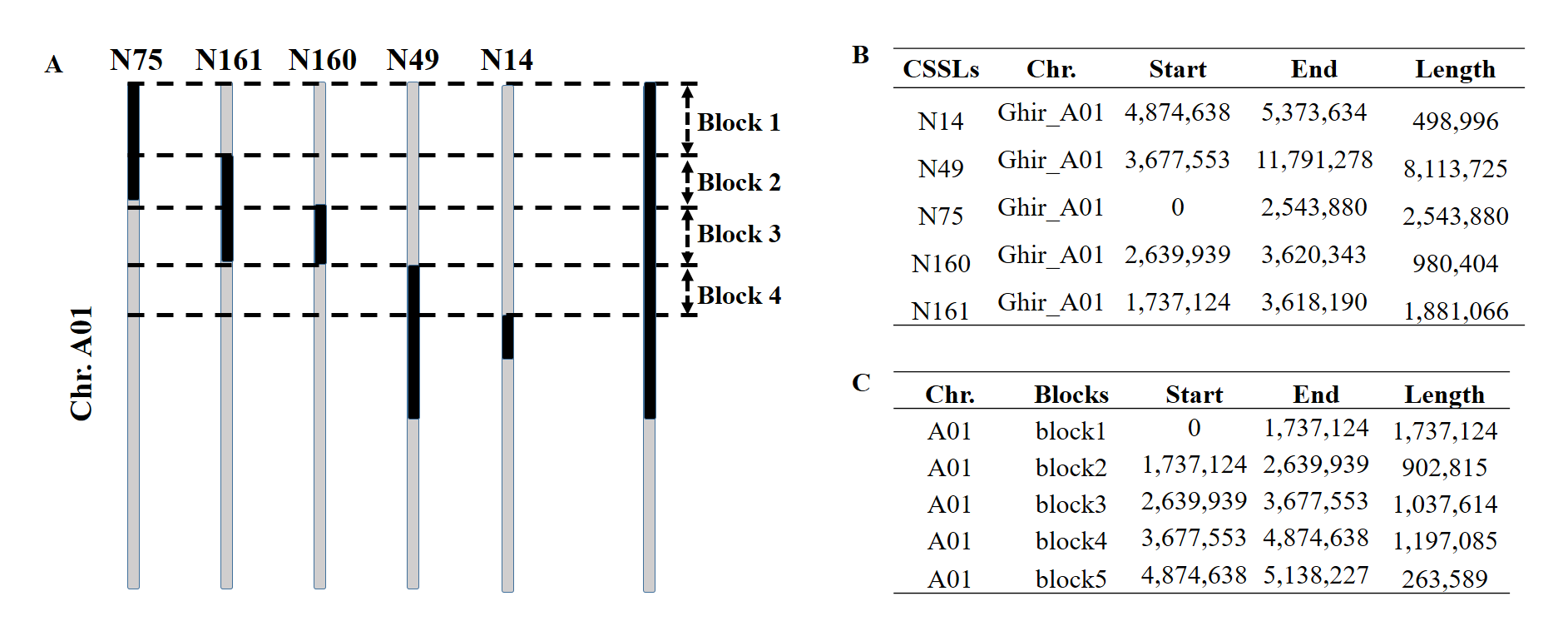

Supplement: Supplementary file 23 — Additional file 23: Figure S10. Example diagram of block partition. (A) The diagram show the principle of block partition; (B) The CSSLs carried the introgression segments on the endpoint of the chromosome A01; (C) First five blocks on the chromosome A01. TIFF [file 12864_2020_6800_MOESM23_ESM.tif]
